# Assessment of *de novo *assemblers for draft genomes: a case study with fungal genomes

**DOI:** 10.1186/1471-2164-15-S9-S10

**Published:** 2014-12-08

**Authors:** Mostafa M Abbas, Qutaibah M Malluhi, Ponnuraman Balakrishnan

**Affiliations:** 1 KINDI Center for Computing Research, College of Engineering, Qatar University, P.O. Box 2713 , Doha, Qatar

## Abstract

**Background:**

Recently, large bio-projects dealing with the release of different genomes have transpired. Most of these projects use next-generation sequencing platforms. As a consequence, many *de novo *assembly tools have evolved to assemble the reads generated by these platforms. Each tool has its own inherent advantages and disadvantages, which make the selection of an appropriate tool a challenging task.

**Results:**

We have evaluated the performance of frequently used *de novo *assemblers namely ABySS, IDBA-UD, Minia, SOAP, SPAdes, Sparse, and Velvet. These assemblers are assessed based on their output quality during the assembly process conducted over fungal data. We compared the performance of these assemblers by considering both computational as well as quality metrics. By analyzing these performance metrics, the assemblers are ranked and a procedure for choosing the candidate assembler is illustrated.

**Conclusions:**

In this study, we propose an assessment method for the selection of *de novo *assemblers by considering their computational as well as quality metrics at the draft genome level. We divide the quality metrics into three groups: g1 measures the goodness of the assemblies, g2 measures the problems of the assemblies, and g3 measures the conservation elements in the assemblies. Our results demonstrate that the assemblers ABySS and IDBA-UD exhibit a good performance for the studied data from fungal genomes in terms of running time, memory, and quality. The results suggest that whole genome shotgun sequencing projects should make use of different assemblers by considering their merits.

## Background

Whole Genome Shotgun (WGS) sequencing projects have been receiving recent attention since it is a critical step in many applications. For example, WGS sequencing of fungi is a fundamental process in several agricultural, environmental, industrial and medical applications [1-3]. Earlier WGS sequencing projects used Sanger sequencing as a central methodology for assembly. With the advent of Next- Generation Sequencing (NGS), recent WGS sequencing projects started to use NGS technologies such as Illumina, Roche 454, etc. These NGS technologies produce a massive amount of reads due to the fact that they have shorter read lengths than their counterpart Sanger sequencing. This massive amount of reads obviously demands high-end computational resources for assembly.

Several assemblers [4-19] have been developed to handle these vast volumes of data. These assemblers have different running time and memory requirements; and produce assembly results with varying quality. For a given dataset, choosing an appropriate assembler is a challenging task that entails the identification of a good trade-off between run time, memory and quality parameters of the assemblers. We can find plenty of recent research works that focus on comparing and evaluating different NGS assemblers in the literature. In [20-25], the evaluation of assemblers considers some quality metrics (which include contiguity, consistency, and accuracy of output assemblies), while [26-28] additionally consider the running-time and memory metrics in their evaluation.

The Assemblathon [20] is a competitive assessment study that evaluates the assembling capabilities of assemblers based on more than 100 metrics. GAGE [21] evaluates the leading assemblers by conducting several assembly experiments that use four different datasets generated using the Illumina technique, three datasets with reference genomes and one dataset without a reference genome. GAGE-B [22] evaluated different finished bacterial genomes based on a set of metrics introduced in GAGE and added new metrics to the evaluation. Finotello et al. [23] explain the pros and cons of assemblers on bacterial data extracted using the 454 pyrosequencing technique. In [24], the authors developed an assessment tool that can be used for evaluating the assemblers either with or without reference genomes. The work in [25], presents a comparative study for four assembly tools to guide the biologists using fungal data generated by the Illumina platform.

To the best of our knowledge, there is no separate evaluation study to assess the performance of *de novo *assemblers for draft genomes in the literature. Hence, this paper proposes a methodology to evaluate *de novo *assembler tools, to assess the capabilities of assemblers based on their computational and quality parameters for draft genomes. The proposed approach is applied on the output of seven assemblers against five fungal pathogens. It classifies the assemblers based on different metrics and identifies suitable assemblers that have a good trade-off between computational and quality performance.

The rest of this paper is organized as follows: the methodology to assess the assemblers and the ranking procedure used in this study is detailed, followed by results and finally, the results are discussed based on different criteria and draws the conclusions.

## Methods

### Evaluation method

In our study, we evaluate the assemblers based on their assembly performance parameters, which are divided into computational as well as quality parameters. The quality metrics that represent the quality of an assembler output generally include contiguity, consistency, and accuracy. Since we do not have the reference genome, we propose an evaluation method for the quality of assemblies which depends on splitting the quality metrics into three groups:

1. Group-1 (g1) measures the level of goodness in the assemblies. This group is called the *goodness group*.

2. Group-2 (g2) measures the level of problems in the assemblies. This group is called the *problems group*.

3. Group-3 (g3) measures the conservation elements in the assemblies. This group is called the *conservation group*.

The above method gives a general prototype for assessing draft genomes. The conservation group metrics may vary based on the type of organism. We can select suitable conservation elements based on the type of organism by hinging on available literature such as [29-33]. In this work, we apply our method on fungal genomes. Therefore, we use the core eukaryotic genes as elements for the conservation group, which can also be used for other higher eukaryotic organisms [33].

After obtaining the computational and quality metrics, we rank the assemblers using a dense ranking technique. Each assembler on a specific dataset takes computational and quality ranks. The rank for each metric is given based on relative performance such that the assembler with the best performance has a rank of 1, the second best has a rank of 2 and so on.

Since measuring the quality of assemblies depends on several metrics and groups we can generally calculate the quality rank for a specific assembler on a specific dataset as follows:

1. Rank the assembler based on each metric individually using the above method.

2. For each metric *m_i_*, assign weight *w_i _*that measures the impact of this metric in its group.

3. Calculate the group rank, $R_{j}$, based on the metric ranks, *r_i_*, and metric weights *w_i _*of this group, using the equation: $R_{j}=\frac{\sum_{i=1}^{n}w_{i}r_{i}}{n}$, where *n *is the number of metrics in this group and *i *= 1, 2,...,*n*.

4. For each group *g_j_*, we assign weight *W_j _*that measures the impact of this group on the quality of assemblies.

5. Calculate the quality rank, *R*, based on the group ranks, *R_j_*, and the group weights *W_j _*by the equation: $R=\frac{\sum_{j=1}^{m}W_{j}R_{j}}{m}$, where *m *is the number of groups and *j *= 1, 2,...,*m*.

For simplicity, we assume that the g1 and g2 metrics and all groups have the same unit weight. We give each metric in g3 a weight proportional to the conservation level. The quality metrics are obtained for all studied assemblers and the current version of draft genome (df_1), which is downloaded from the WGS project page in NCBI (http://www.ncbi.nlm.nih.gov/bioproject/). Also, the quality metrics are measured at both the contigs and scaffolds levels. We use the quality rank for the contigs level as the quality rank of the assemblies.

### Evaluation metrics

For computational performance, we consider the running time and memory consumption metrics. The running time is the total time taken by the assembler to complete the assembly process for a given dataset, whereas memory consumption is the maximum amount of memory used during the entire execution period. Time and memory measurements are taken using the Linux utility commands *time *and *top *respectively. For quality performance, we consider the following metrics:

**Largest contig size: **The length of the largest contig in an assembly.

**N50 size: **The length of the smallest contig *x*, which makes the ratio of cumulative length of contigs from this length *x *to largest contig size covers at least 50% of the bases of the assembly. An assembler with high N50 size value is obviously considered to be a high quality assembler.

**L50: **The number of contigs with length larger than or equal to N50.

**Chaff bases percentage **[21]: The percentage ratio of cumulative length of chaff contigs to cumulative length of all contigs in the assembly, where a chaff contig is a single contig with a length less than 200 bp [21]. The high percentage of chaff contigs length leads to problems in further genomic analysis [21]. Hence, high quality assemblers should possess low chaff bases percentage.

**Number of N's: **The total number of uncalled bases or gaps (N's) in the assembly bases. Mis-assemblies and gaps usually result from repeats as well as secondary structures, either in unrepresented GC-rich regions or in un-sequenced regions due to a low depth sequence coverage [34]. This value is high for low quality assemblies.

**CEGs percentages: **The percentages of different Core Eukaryotic Genes (CEGs) mapped in the assemblies. In [33], the authors identify 248 Core Eukaryotic Genes (CEGs), which are highly conserved, present in low copy numbers in higher eukaryotes, and can be used in describing the gene space. Based on the average degree of conservation observed from each CEG, the work in [33] divides the CEGs into four groups (group 1 has the least conserved CEGs while group 4 has the most conserved CEGs). This work demonstrates that the percentage of CEGs can be useful as a complement for the metrics of N50 size and x-fold coverage. In our study we consider the percentage of CEGs in each group completely mapped from the assemblies as an independent metric. In other words, we consider four metrics csg1%, csg2%, csg3% and csg4%, which represent the percentage of CEGs in the groups 1, 2, 3, and 4 (defined in [33]) respectively. Refereeing to the values (defined in supplementary table S4 of [33]) to determine the conservation degree of each group, we assume that, the weights of metrics csg1%, csg2%, csg3% and csg4% are 0.76, 0.92, 1.04, and 1.28 respectively.

We split these metrics into our three groups as follows:

• The *goodness group*, g1, contains the metrics (largest contig size, N50 size and L50), which reflect the assembly connectivity and the nature of the bulk of the assembly [35].

• The *problems group*, g2, contains the metrics chaff bases percentage and No. of N's.

• The *conservation group*, g3, contains the metrics csg1%, csg2%, csg3% and csg4%, which represent in our case (fungal genomes) conservation elements in the assemblies. These metrics can be used for the other higher eukaryotic genomes [33].

All these quality metrics are measured using the QUAST assessment tool [24] except CEGs percentages, which are calculated using CEGMA tool [33,36]. The g1 metrics and No. of N's metric are calculated after removing any chaff contig in the assemblies.

### Fungal species data

To apply our assessment method, we have chosen five fungal pathogens from several WGS sequencing projects released in 2013, which are still in the draft genome level and are missing the reference genome (Table 1) [37-41]. Additionally, all datasets are generated by the Illumina HiSeq 2000 sequencing platform with paired-end layout and are downloaded from the DNA Data Bank of Japan (DDBJ, http://www.ddbj.nig.ac.jp/). The read length for each data is 100 bp. Since the size of 90% of available fungal data is less than 60 Mb [42], we have chosen four datasets with a size less than 60 Mb and one dataset with a size above this threshold (see Table 1).

**Table 1:** List of fungal genomes studied in the experiments.

Species	Accession Number in DDBJ/EMBL/GenBank	SRA Accession Number in NCBI	Estimated Size (Mbp)	Estimated GC content %	Data Size (GB)
Botryotinia fuckeliana (BcDW1)[37]	AORW00000000	SRR680162	42.1323	42	23

Neofusicoccum parvum (UCRNP2)[38]	AORE00000000	SRR654031	42.5928	56.8	24

Togninia minima (UCRPA7)[39]	AORD00000000	SRR654175	47.4654	49.7	18

Eutypa lata (UCREL1)[40]	AORF00000000	SRR654028	54.0058	46.6	23

Puccinia striiformis f. sp. tritici (PST21)[41]	AORR00000000	SRR653741	73.0475	44.4	13

### Assemblers

For assembling the above chosen fungal datasets, we selected seven open source assemblers, which can handle the short reads produced by NGS platforms: ABySS [11], IDBA-UD [17], Minia [16], SOAP [5], SPAdes [19], Sparse [18], and Velvet [7]. For Minia, we extract the assembly only at the contigs level (available level in the used version), whereas for all other assemblers, we extract the assemblies at both contigs and scaffolds levels. Table 2 summarizes the details of assemblers that are used in this study including their websites, and versions.

**Table 2:** List of assemblers evaluated in the study.

Assembler	Website	Version
ABySS	http://www.bcgsc.ca/platform/bioinfo/software/abyss	1.3.7

IDBA-UD	http://i.cs.hku.hk/~alse/hkubrg/projects/idba_ud/	1.0.9

Minia	http://minia.genouest.org/	1.5901

SOAPdenovo(SOAP)	http://soap.genomics.org.cn/soapdenovo.html	2-r240

SPAdes	http://bioinf.spbau.ru/spades	3.0.0

SparseAssembler(Sparse)	http://sourceforge.net/projects/sparseassembler/	--

Velvet	https://www.ebi.ac.uk/~zerbino/velvet/	1.2.10

### Experimental setup

All assembly experiments of the five datasets over the seven assemblers are conducted on a dual Octa-core processors (2.9 GHz Intel Xeon E5-2690) machine with 128 Gb RAM. All experiments are conducted using a single core. Additionally, we run the assembler tools several times (except for IDBA-UD and SPAdes) with different k-mer parameter and choose the optimal value for k that exhibits high quality of g1 and g2 metrics. Since the IDBA-UD and SPAdes assemblers are iterative in nature, the k-mer value that exhibits best quality metrics are implicitly chosen as an optimal k-mer from multiple k-mer values so we use the default k-mer values as defined in the tool.

## Results

In this study, we focus on assessing the parameters that influence the selection of *de novo *assembler for assembling a given dataset. For that, the assembling experiments are conducted and the computational as well as quality metrics are measured. In this section, we analyze and discuss these metrics for all the seven assemblers.

### Computational time performance

In general, the running time of a given dataset is a parameter in deciding the candidate assembler. Hence, we measure the running time of seven assemblers over the five datasets (see Table 3). We rank the assemblers based on their running time to decide the candidate assemblers. The ranks for different datasets are shown in the table inside parenthesis. From Table 3 Minia has the best rank in three datasets which have larger estimated genome size. Among the assemblers that have both contigs and scaffolds levels, Velvet obtains the best rank in four datasets while SPAdes obtains the worst rank in all datasets. The running time of Minai and Velvet for any dataset did not exceed 10% of the running time of SPAdes for the same dataset.

**Table 3:** Running time measurement in hours of the seven assemblers against the five fungal pathogens.

Dataset	ABySS	IDBA-UD	Minia	SOAP	SPAdes	Sparse	Velvet
BcDw1	2.43(5)	7.65(6)	1.02(2)	0.89(1)	17.41(7)	1.24(4)	1.02(2)

UCRNP2	3.45(5)	9.64(6)	2.18(3)	1.55(2)	32.49(7)	2.21(4)	1.5(1)

UCRPA7	2.21(5)	5.96(6)	1.11(1)	1.96(4)	13.51(7)	1.45(3)	1.28(2)

UCREL1	3.41(5)	8.25(6)	1.3(1)	2.12(4)	21.63(7)	1.87(3)	1.71(2)

PST21	3.89(5)	7.88(6)	1.25(1)	2.73(4)	-------	1.51(3)	1.46(2)

Parentheses contain the rank the assembler on the specific dataset. "---" Indicates a corrupted assembly process.

### Memory consumption performance

The most critical parameter in selecting a candidate assembler tool is the memory needed by the tool during the assembly process, especially with the increase of data volumes in NGS platforms. An assembler demanding huge memory can be the reason for excluding it from the list of candidate assemblers. Table 4 gives the measured maximum memory usage of all the seven assemblers over the five datasets. We rank the assemblers based on their memory consumption to decide the candidate assemblers. The rank for a given dataset is calculated and given inside parenthesis. From Table 4 Minia obtains the best rank among all datasets followed by Sparse, while SPAdes obtains the worst rank for all datasets. The maximum memory consumption of Minia and Sparse for any dataset did not exceed 1% and 6%, respectively, of the maximum memory consumption of SPAdes for the same dataset. In the case of PST21 dataset, the SPAdes assembler takes more than 128 Gb (the maximum available memory in our machine).

**Table 4:** Memory consumption in GB of the seven assemblers against the five fungal pathogenes.

Dataset	ABySS	IDBA-UD	Minia	SOAP	SPAdes	Sparse	Velvet
BcDw1	5.47(3)	5.91(4)	0.20(1)	22.2(6)	42(7)	1.68(2)	18.3(5)

UCRNP2	8.86(3)	12.1(4)	0.28(1)	19.2(5)	49.4(7)	2.54(2)	28.5(6)

UCRPA7	5.79(3)	6.89(4)	0.19(1)	27.7(6)	36.9(7)	1.99(2)	23.2(5)

UCREL1	5.98(3)	7.37(4)	0.18(1)	27(6)	46(7)	1.83(2)	22.3(5)

PST21	19.5(4)	10.6(3)	0.31(1)	36.6(6)	------	2.69(2)	30.9(5)

Parentheses contain the rank the assembler on the specific dataset. "---" indicates a corrupted assembly process.

### Assembling quality

The quality metrics of an assembler play an important role in the selection of candidate assembler. For a given dataset, various quality metrics of each assembler at the contigs as well as scaffolds level are demonstrated (Tables S1-S5; Additional file 1 and Figs. S1-S5; Additional file 2).

**Figure 1:**
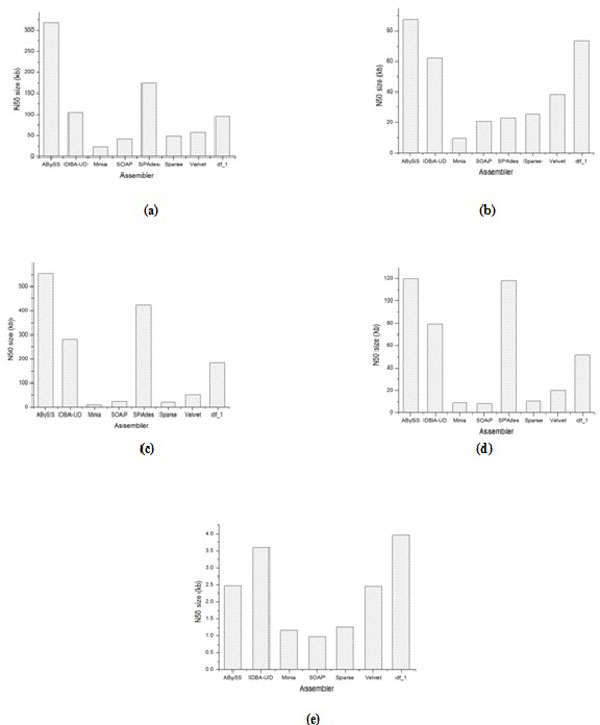
**Comparison for N50 size metric for the studied assemblers at contigs level**. (a): BcDw1, (b): UCRNP2, (c): UCRPA7, (d): UCREL1, (e): PST21.

In (Table-S1; Additional file 1), which gives the quality metrics of all assemblers for the BcDw1 dataset, the assemblers ABySS, IDBA-UD, and SPAdes have better goodness (g1) metrics performance than the current draft genome (df_1) at the contigs level, while the assemblers ABySS, IDBA-UD, and Velvet have better g1 metrics performance than df_1 at the scaffolds level (see Fig. S1; Additional file 2). For example, the assemblies of ABySS, IDBA-UD, and SPAdes have superior N50 size (see Figure 1(a)). Based on the g1 metrics at the contigs level, ABySS is the highest quality assembler, whereas Minia is the lowest quality assembler for the BcDw1 dataset. Similarly for g1 metrics, IDBA-UD is the highest quality assembler, whereas SOAP is the lowest quality assembler at the scaffolds level. Furthermore, SOAP obtains consistent quality at both contigs as well as scaffolds levels. Sparse generates the large percentage of chaff bases length at both contigs and scaffolds levels, which makes it a low quality assembler in g2 metrics. There are no gaps at contigs level for all assemblers except ABySS. Velvet, on the other hand, produced a huge number of gaps with respect to other assemblers at the scaffolds level. Based on the g2 metrics, at the scaffolds level, IDBA-UD and SPAdes are high quality assemblers, whereas ABySS, Sparse, and Velvet become low quality assemblers from the problems (g2) metrics point of view. At the contigs level, the ABySS, IDBA-UD, and Velvet assemblers have better conservation metrics (g3) rank with respect to other assemblers followed by SOAP. While at the scaffolds level, SOAP has better g3 rank followed by SPAdes. Overall, SPAdes and IDBA-UD have the best quality ranks at the contigs and scaffolds level, respectively (see Tables 5, 6).

**Table 5:** Quality results of all assemblies against the five fungal pathogens at the contigs level.

Dataset	ABySS	IDBA-UD	Minia	SOAP	SPAdes	Sparse	Velvet	df_1
BcDw1	1.97	1.92	4.42	3.99	1.87	3.91	2.64	2.18

UCRNP2	2.39	1.61	4.69	3.73	2.44	3.81	2.52	1.89

UCRPA7	1.95	1.75	5.24	3.88	1.48	4.58	3.16	2.17

UCREL1	2.21	1.84	5.11	5.01	1.85	4.87	3.14	2.75

PST21	3.27	1.76	4.64	5.58	------	4.28	3.08	1.38

**Table 6:** Quality results of all assemblies against the five fungal pathogens at the scaffolds level.

Dataset	ABySS	IDBA-UD	SOAP	SPAdes	Sparse	Velvet	df_1
BcDw1	2.65	1.71	3.63	2.74	3.96	2.98	3.07

UCRNP2	2.77	2	3.73	3.66	3.7	3.19	2.2

UCRPA7	2.75	1.35	4.37	2.13	4.43	3.43	3.27

UCREL1	3.43	1.53	4.75	2.57	4.23	2.42	3.43

PST21	3.38	1.86	3.85	------	2.86	2.46	------

In the assembly of the UCRNP2 dataset (see Table-S2; Additional file 1), ABySS and IDBA-UD show higher g1 quality performance as compared to the current draft genome at both contigs and scaffolds levels (see Fig. S2; Additional file 2). At the contigs level, ABySS has superior N50 size (see Figure 1(b)). Based on the g1 metrics at the contigs level, ABySS is a high quality assembler, whereas Minia is a low quality assembler for the UCRNP2 dataset. In addition, IDBA-UD is the high quality assembler for g1 at the scaffolds level. Furthermore, ABySS, Minia, and Sparse (except Minia at scaffolds level) generate a huge percentage of chaff bases length 23%, 54%, and 66%, respectively, at both the contigs as well as scaffolds levels, which makes them as low quality assemblers from g2 metrics point of view. ABySS, IDBA-UD, SPAdes, and Velvet produce a large numbers of gaps with respect to other assemblers at scaffolds level. Based on g2 metrics, IDBA-UD is the high g2 quality assembler, while ABySS and Sparse are low g2 quality assemblers, at the contigs level. On the other hand, at the scaffolds level, SOAP proves to be the best g2 quality assembler whose output is as good as the draft genome, while ABySS and Sparse are low g2 quality assemblers. In addition, Velvet proves to be the highest g3 quality assembler preserving the highest percentage of CEGs in three conservation groups at both contigs as well as scaffolds levels. Overall, IDBA-UD has the best quality rank at both the contigs and scaffolds levels (see Tables 5, 6).

In the assembly of UCRPA7 dataset (see Table-S3; Additional file 1), the g1 quality of ABySS, IDBA-UD, and SPAdes is better than the current draft genome (df_1) at the contigs level, while ABySS, IDBA-UD, SPAdes and Velvet have better g1 metrics performance than the df_1 at scaffolds level (see Fig. S3; Additional file 2). Based on the g1 metrics at contigs level, ABySS is the high quality assembler, whereas Minia is the low quality assembler for the UCRPA7 dataset. In contrast, at the scaffolds level, the IDBA-UD assembler shows best g1 metrics quality performance whereas SOAP demonstrates the worst g1 quality. The g1 metrics for SPAdes are approximately equal in both the contigs and scaffolds levels. At the contigs level, ABySS, IDBA-UD, and SPAdes have superior N50 size (see Figure 1(c)). By considering the g2 quality metrics, Sparse generates the largest percentage of chaff bases length as compared to other assemblers at both the contigs and scaffolds levels. There are no gaps at the contigs level for all assemblers except ABySS. Velvet produces a huge number of gaps at the scaffolds level. Regarding g3 quality metrics, the IDBA-UD, and SPAdes are high quality assemblers at both contigs and scaffolds levels. SPAdes and IDBA-UD have the best quality rank at the contigs and scaffolds levels, respectively (see Tables 5, 6).

In the assembly of UCREL1 dataset (see Table-S4; Additional file 1), ABySS, IDBA-UD, and SPAdes exhibit high quality g1 metrics performance, which is better than the performance of the current draft genome (df_1) at the contigs level, while ABySS, IDBA-UD, SPAdes and Velvet have better g1 metrics performance than df_1 at the scaffolds level (see Fig. S4; Additional file 2). At the contigs level, the ABySS assembler shows the best g1 quality metrics whereas, IDBA-UD shows best g1 quality metrics performance at the scaffolds level. At the contigs level, ABySS, IDBA-UD, and SPAdes demonstrate high N50 size (see Figure 1(d)). By considering the g2 quality metrics, Sparse generates the largest percentage of chaff bases length at both the contigs and scaffolds levels with respect to other assemblers. There are no gaps at the contigs level for all assemblers except ABySS. Velvet produces a huge number of gaps at the scaffolds level. Regarding g3 quality metrics, the IDBA-UD is the best quality assembler at the contigs level while IDBA-UD and Velvet are the best quality assemblers at the scaffolds level. IDBA-UD has the best quality rank at both the contigs and scaffolds levels (see Tables 5, 6).

In the assembly of PST21 dataset (Table-S5; Additional file 1), IDBA-UD exhibits better g1 metrics performance than the other assemblers, which is lower quality than the current draft genome at the contigs level (see Fig. S5; Additional file 2). At the scaffolds level, Velvet produces the best g1 quality metrics for the assemblies of the PST21 dataset. There is no scaffold level submitted for the current draft genome for PST21 dataset. At the contigs level, ABySS, IDBA-UD, and Velvet demonstrate high N50 size (see Figure 1(e)). Considering g2 quality metrics, all assemblers except IDBA-UD and Velvet generated large percentages of chaff bases length at the contigs level. ABySS shows a less efficient chaff bases metric at the scaffolds level. ABySS, SOAP and Velvet produce a huge number of gaps at the scaffolds level. Considering g3 quality metrics, ABySS is the best quality assembler at the contigs level (but demonstrates significantly lower performance than the current draft genome), while Sparse is the best quality assembler at the scaffolds level. IDBA-UD has the best quality rank at both the contigs and scaffolds levels (see Tables 5, 6).

## Discussion and conclusions

In this section, the studied assemblers are divided into classes based on the parameters obtained from the above experiments, which include running time, memory consumption, and quality. The K-means clustering method [43] is employed to classify the assemblers into these classes using the SPSS (Statistical Package for Social Sciences) tool. Next, we identify the assemblers that have a good trade-off between running time, memory consumption, and quality, and therefore can be selected as the candidate assemblers. Finally, we preset the conclusions of our study.

Table 7 shows the running time, memory consumption, and quality classes for each assembler based the partitioning process. Though Minia is classified as a low-quality assembler, the quality of assemblies for Minia gets enhanced as the expected genome size increases. The ideal assemblers achieve an ideal trade-off between running time, memory consumption, and quality of assemblies (i.e., the assemblers that belong to the fastest class, most memory-efficient class, and high-quality class). Unfortunately, among the studied assemblers, we cannot identify such an ideal assembler. However, ABySS and IDBA-UD have a good trade-off between running time, memory utilization, and quality of assemblies as both ABySS and IDBA-UD belong to the medium-fast, memory-efficient and high-quality classes.

**Table 7:** Classification of the seven assemblers based on computational and quality metrics.

Assembler	Running time class	Memory consumption class	Quality class
ABySS	medium-fast	memory-efficient	high-quality

IDBA-UD	medium-fast	memory-efficient	high-quality

Minia	fastest	most memory-efficient	low quality

SOAP	fastest	less memory-efficient	medium-quality

SPAdes	Slow	memory-inefficient	high-quality

Sparse	fastest	most memory-efficient	medium-quality

Velvet	fastest	less memory-efficient	high medium-quality

**Figure 2:**
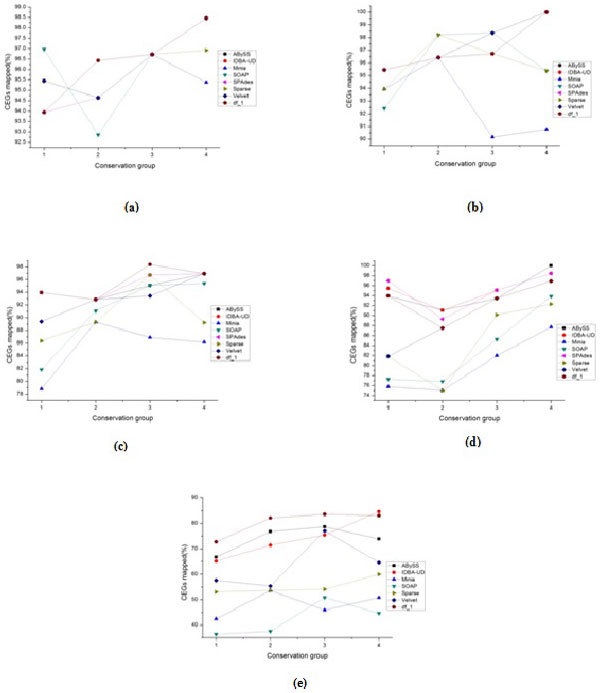
**Percentage of CEGs in four conservation groups for all assemblies at contigs level**. CEGs Mapping results of the seven assemblers outputs and the current draft genome(df_1) at contigs level for the studied datasets in the four groups of core genes. (a): BcDw1, (b): UCRNP2, (c): UCRPA7, (d): UCREL1, (e): PST21.

**Figure 3:**
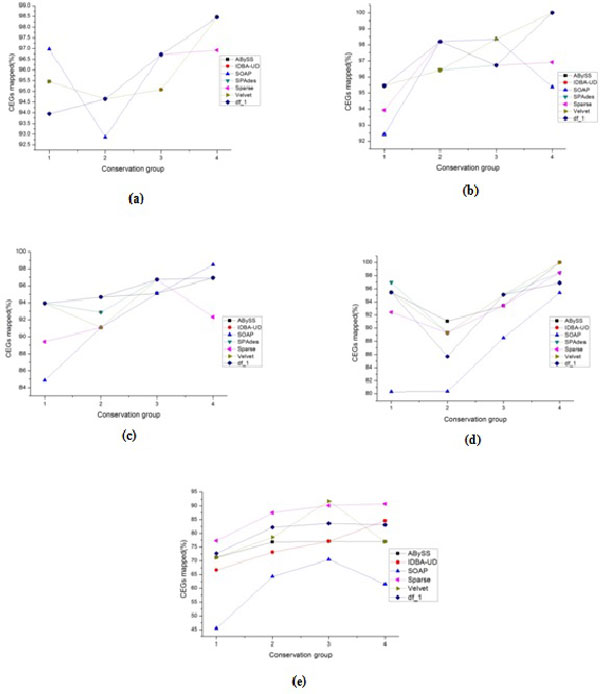
**Percentage of CEGs in four conservation groups for all assemblies at the scaffolds level**. CEGs Mapping results of all assemblers outputs (except Minia) and the current draft genome(df_1) at scaffolds level for the studied datasets in the four groups of core genes. (a): BcDw1, (b): UCRNP2, (c): UCRPA7, (d): UCREL1, (e): PST21. In PST21 dataset, the df_1 represents the assemblies at the contigs level because we do not have scaffolds level.

Considering the quality metrics in details, the behavior of g1 and g3 metrics is approximately similar to the overall quality ranking. For g2 metrics we note that, for all datasets, there are no gaps at the contigs level for all assemblers except ABySS. However ABySS and Velvet produce a large number of gaps for most datasets at the scaffolds level, while Sparse produces a large percentage of chaff bases. Another observation is that though some assemblers may demonstrate similar overall level of preservation of the 248 CEGs, they differ at the individual conservation group percentages of CEGs (see Figures 2, 3 and Tables S1-S5; Additional file 1). This suggests the idea of combining two or more different assemblies for improving the overall quality of the assemblies. IDBA-UD and SPAdes (which are in the high-quality class) use multiple k-mers and capture the benefits of the large k-mer. In [44-47], the authors proposed the usage of different tools in post-assembly to merge different assemblies in order to benefit from the advantages of each one.

In conclusion, this paper proposes a general methodology for assessment of *de novo *assemblers for draft genomes in terms of running time, memory consumption, and quality metrics. The quality metrics are split into three groups: g1 measures the goodness of the assemblies, g2 measures the problems of the assemblies, and g3 measures the conservation elements in the assemblies. We believe that, adding more conservation metrics from closely related species in g3 can enhance the assessment results. We apply our method for assessing seven open source *de novo *assemblers to assemble five fungal pathogens at draft genome level. Based on our results, we partition the studied assemblers into different classes based on the criteria of time, memory, and quality. Our results support the idea of making the assemblies of WGS sequencing projects using different assemblers to exploit the strengths of each one by combining the corresponding assemblies. ABySS and IDBA-UD offer a good trade-off between running time, memory, and quality among the studied assemblers for the studied datasets. The rapidly growing number of WGS sequencing projects can take advantage of our results and proposed methodology to choose an appropriate assembler for best quality assemblies based on available computational resources. The results of this research work are freely available at http://confluence.qu.edu.qa/display/download/bioinf.

## Competing interests

The authors declare that they have no competing interests.

## Authors' contributions

MMA and QMM designed the method; MMA ran the experiments; MMA, QMM and P.B analyzed the results; MMA wrote the draft of the manuscript. All authors read and approved the final manuscript.

## Supplementary Material

Additional file 1 **Main spreadsheet containing all results**. Details of quality metrics and its ranks for every assembly for each dataset. First, second, third, fourth, and fifth sheets contain Tables S1, S2, S3, S4, and S5 which are represent a detailed results of BcDw1, UCRNP2, UCRPA7, UCREL1, and PST21 datasets, respectively. Click here for file

Additional file 2 **Basic plots for all results**. Basic plots for all assemblies of all datasets generated using QUAST tool [24]. For each dataset we have six plots grouped in a single figure: a1, a2, b1, b2, c1 and c2 such that: a1 and a2 represent the cumulative length plots at contigs and scaffolds levels, respectively, b1 and b2 represent the GC-content plots at contigs and scaffolds levels, respectively, c1 and c2 represent the Nx plots for different × values at contigs and scaffolds levels, respectively. Figures S1, S2, S3, S4, and S5 represent the basic plots for BcDw1, UCRNP2, UCRPA7, UCREL1, and PST21 datasets, respectively. Click here for file

## Declarations

The publication costs for this article were funded by the Qatar National Research Fund grant (No. 4-1454-1-233) awarded to QMM.

This article has been published as part of *BMC Genomics *Volume 15 Supplement 9, 2014: Thirteenth International Conference on Bioinformatics (InCoB2014): Computational Biology. The full contents of the supplement are available online at http://www.biomedcentral.com/bmcgenomics/supplements/15/S9.
