# Supplementary material for: Assessment of de novo assemblers for draft genomes: a case study with fungal genomes
Source: BMC Genomics. 2014 Dec 8;15(Suppl 9):S10. doi: 10.1186/1471-2164-15-S9-S10 (PMC4290589; doi:10.1186/1471-2164-15-S9-S10)

This file contains the basic plots for all assemblies of all datasets generated using QUAST tool [24].

For each dataset we have six plots: a1, a2, b1, b2, c1 and c2 such that:

a1: represents the cumulative length at contigs level.

a2: represents the cumulative length at scaffolds level.

b1: represents the GC-content at contigs level.

b2: represents the GC-content at scaffolds level.

c1: represents the Nx at contigs level for different x values.

c2: represents the Nx at scaffolds level for different x values.

Where Nx is defined like N50 by replacing 50 by x.

For each assembler in scaffolds plots, we have two curves: one for the original assemblies and the other for assemblies after splitting continuous fragments of N's of length ≥ 10.

The only considered contigs(scaffolds) here which with length ≥ 200 bp.

##

## Figure S1 - Basic plots for BcDw1 dataset

( 1a)


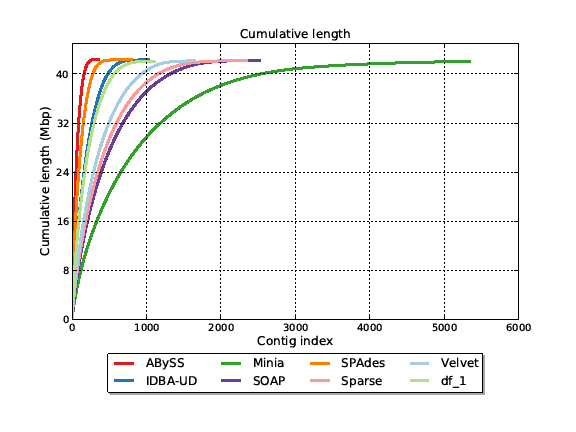


(1b)


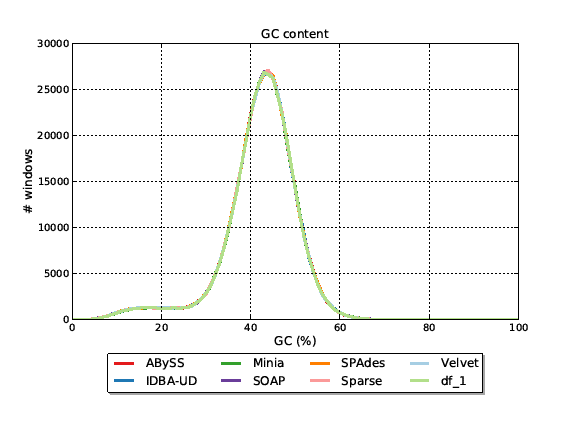


(1c)


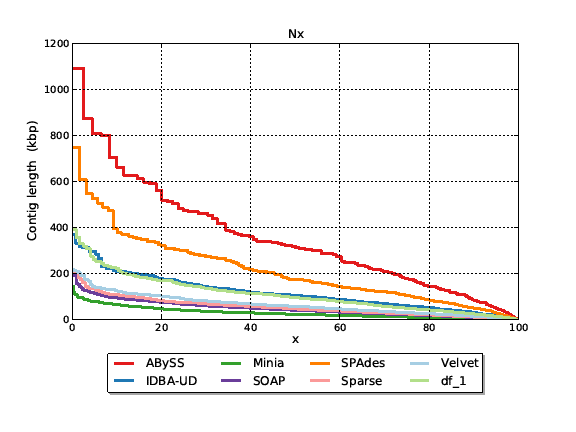


( 2a)


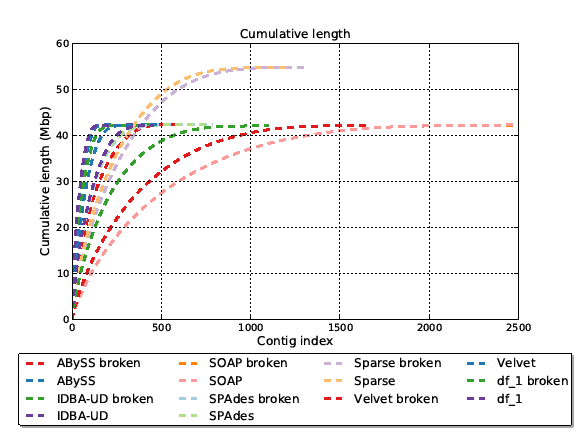


(2b)


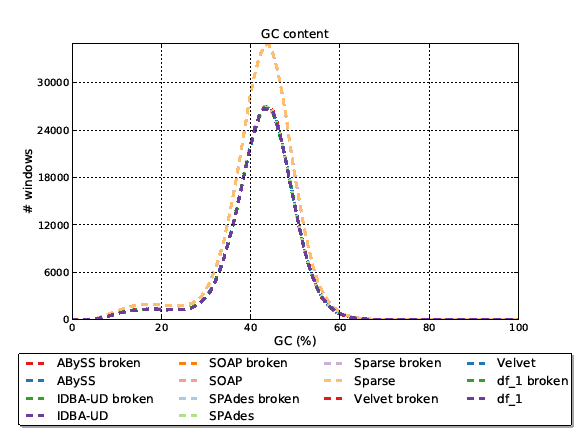


(2c)


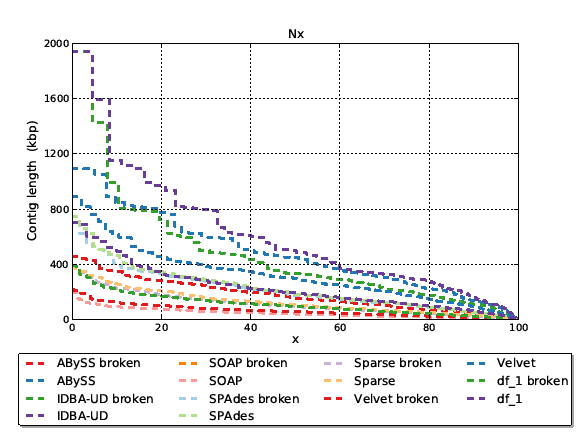


## Figure S2 - Basic plots for UCRNP2 dataset

(1a)


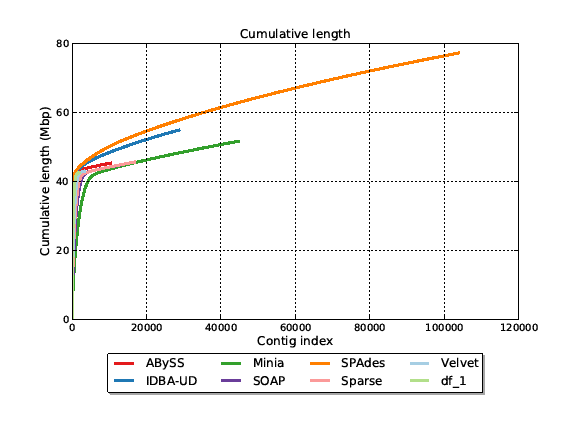


(1b)


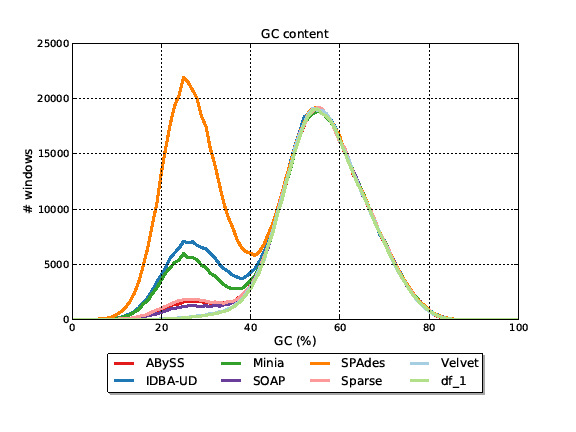


(1c)


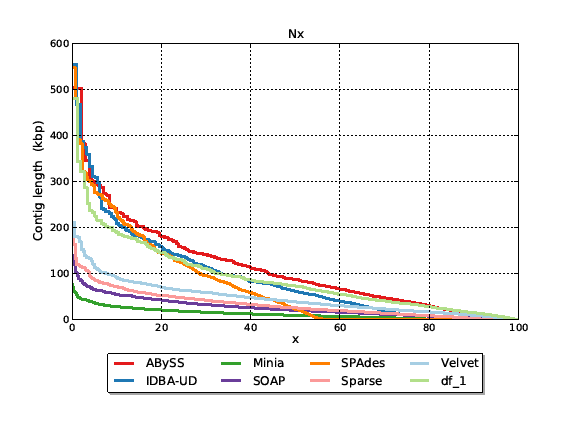


(2a)


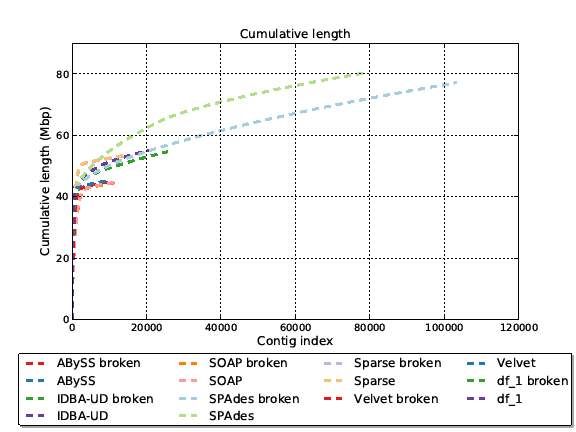


(b2)


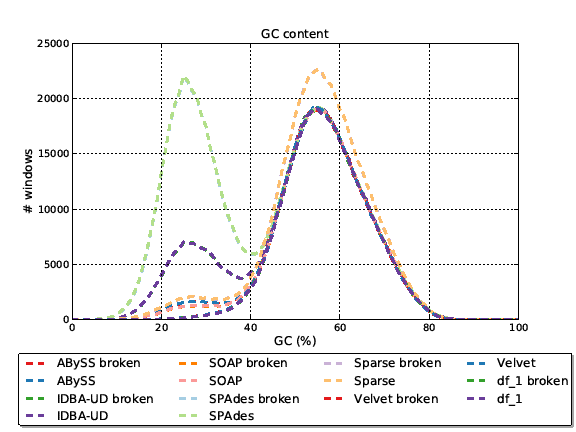


(2c)


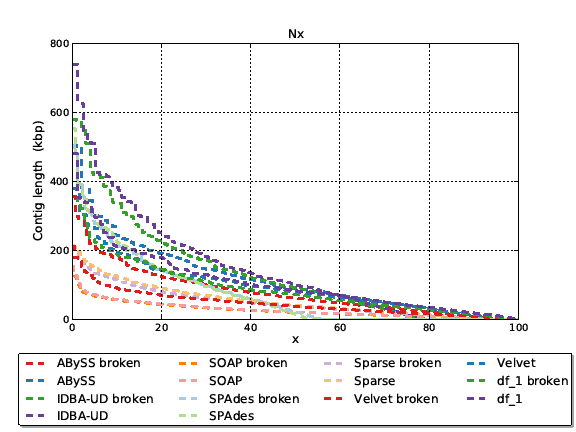


## Figure S3 - Basic plots for UCRPA7 dataset

(1a)


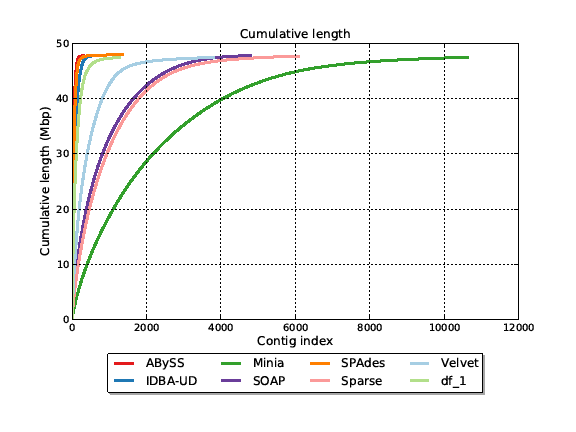


(1b)


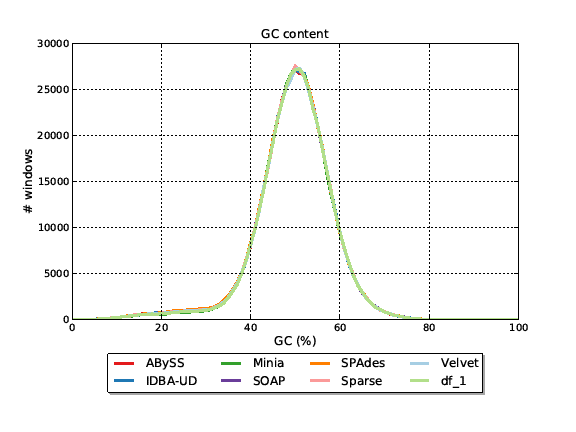


(c1)


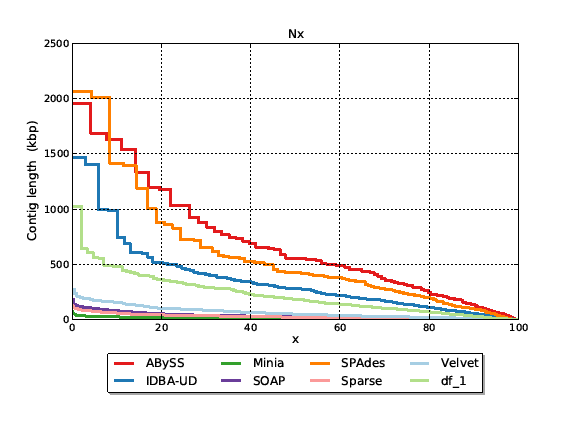


(2a)


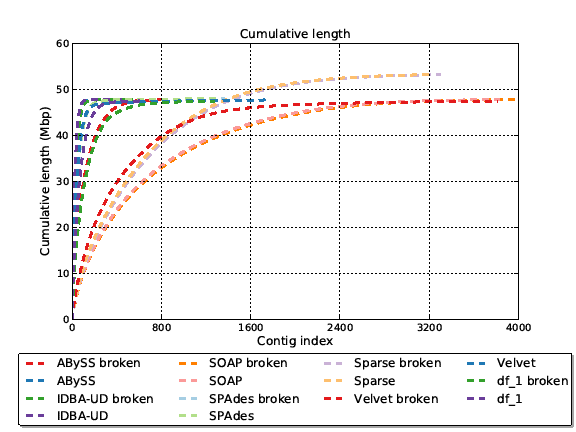


(2b)


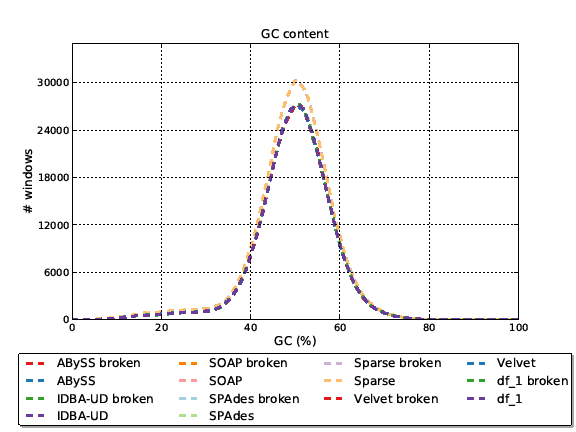


(2c)


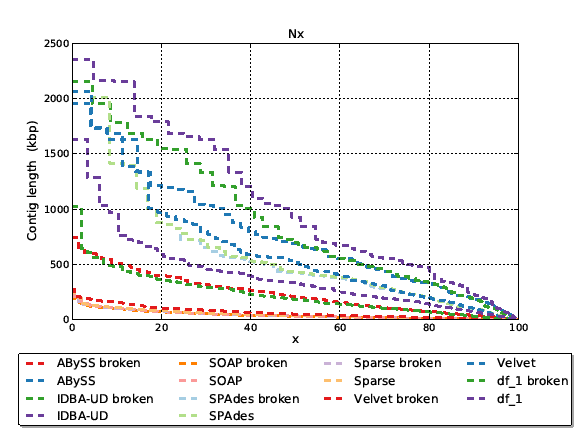


(1a)

(2a)

(1b)

(2b)

(1c)

(2c)

## Figure S4 - Basic plots for UCREL1 dataset

(1a)


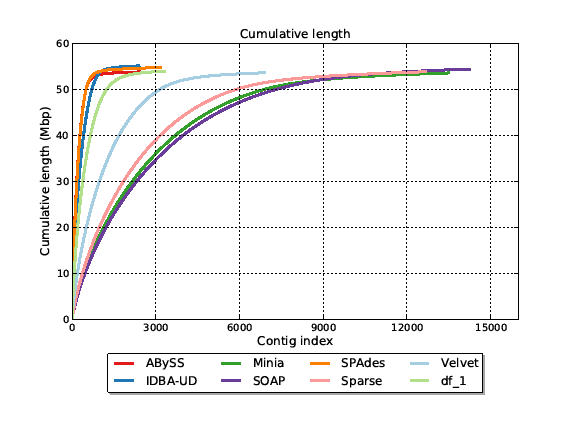


(1b)


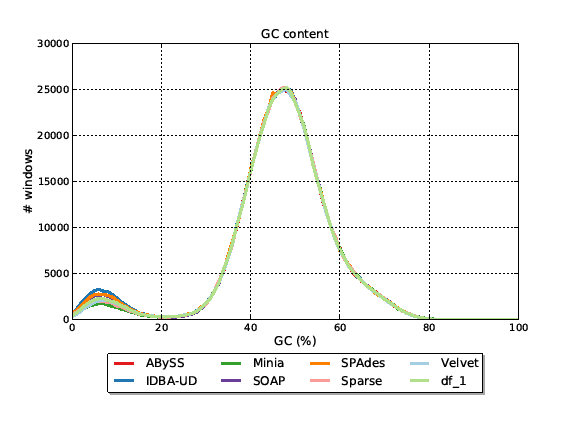


(c1)


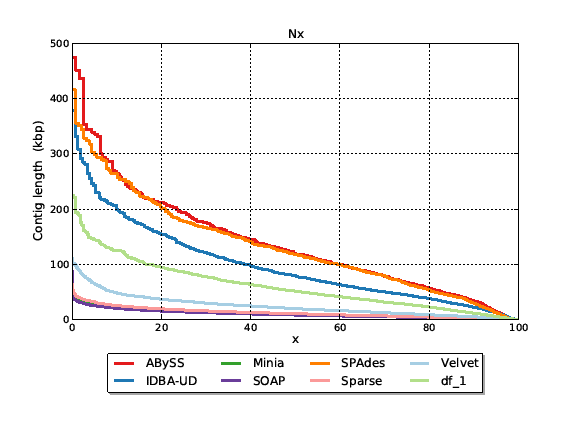


(2a)


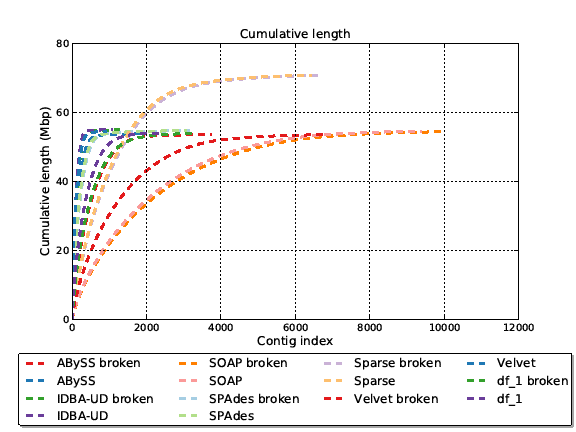


(2b)


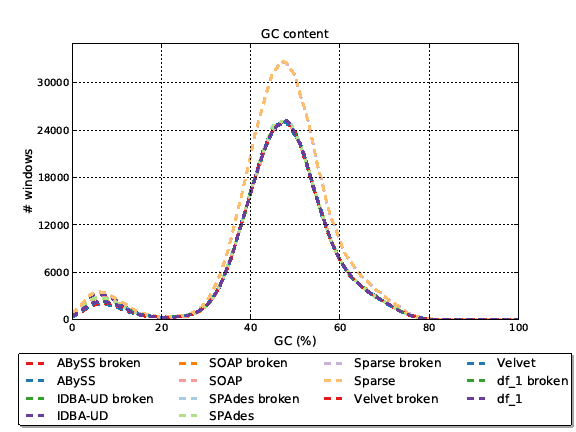


(c2)


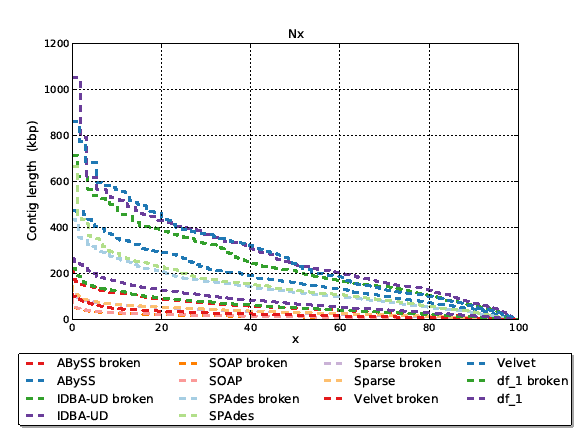


## Figure S5 - Basic plots for PST21 dataset

(a1)


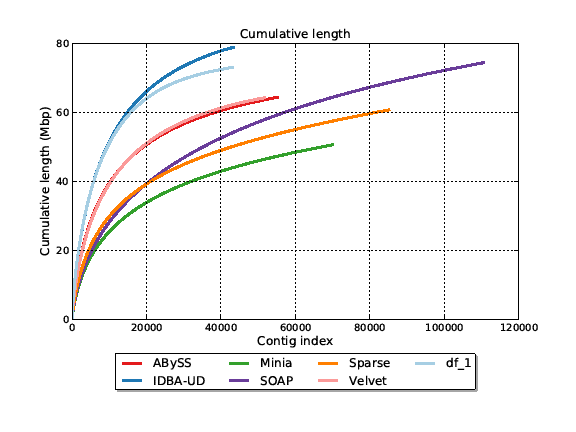


(b1)


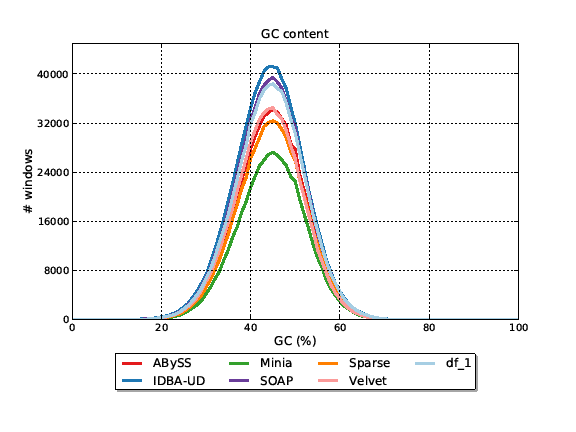


(c1)


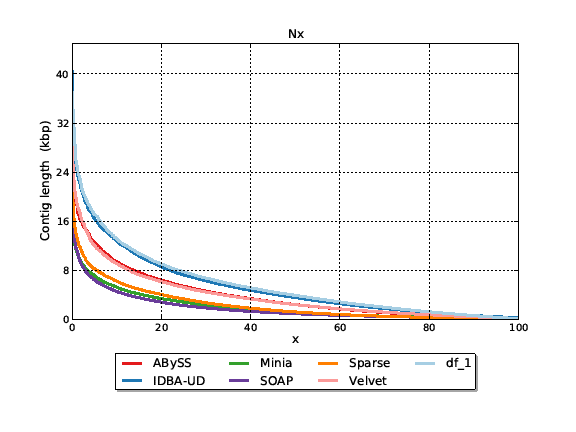


(a2)


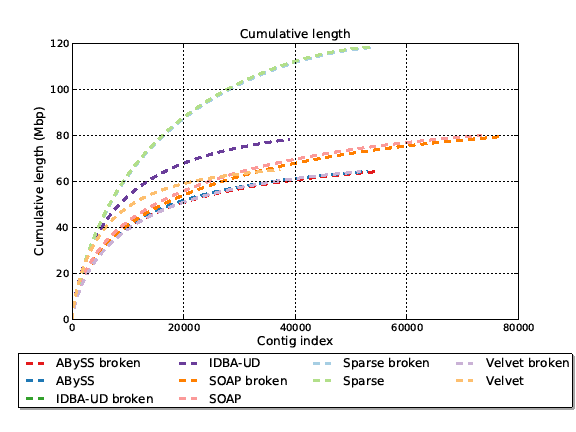


(b2)


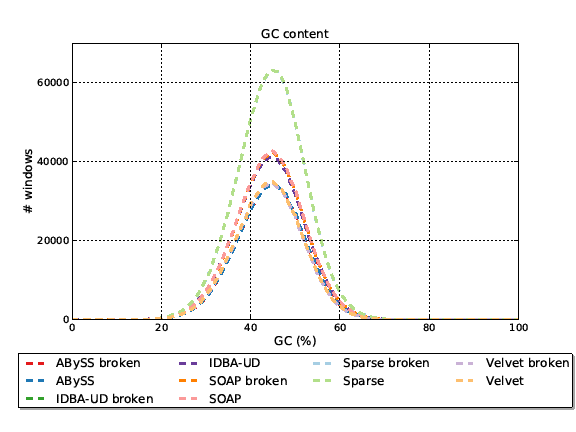


(c2)


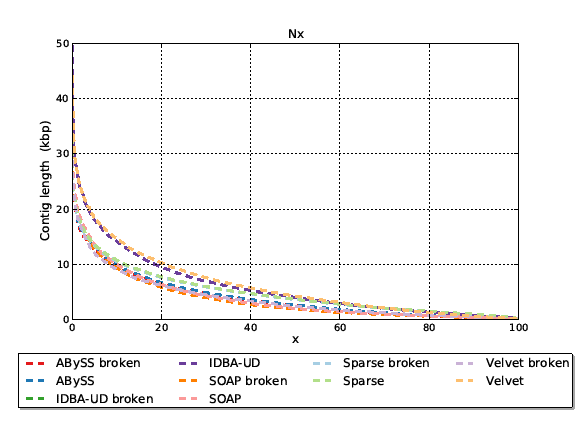

Supplement: Additional file 2 — Basic plots for all results. Basic plots for all assemblies of all datasets generated using QUAST tool [24]. For each dataset we have six plots grouped in a single figure: a1, a2, b1, b2, c1 and c2 such that: a1 and a2 represent the cumulative length plots at contigs and scaffolds levels, respectively, b1 and b2 represent the GC-content plots at contigs and scaffolds levels, respectively, c1 and c2 represent the Nx plots for different × values at contigs and scaffolds levels, respectively. Figures S1, S2, S3, S4, and S5 represent the basic plots for BcDw1, UCRNP2, UCRPA7, UCREL1, and PST21 datasets, respectively. [file 1471-2164-15-S9-S10-S2.docx]
